# Rare case of a massive anterior mediastinal schwannoma causing cardiac compression with concurrent cervical spine lesion causing spinal cord compression

**DOI:** 10.1186/s13019-025-03452-4

**Published:** 2025-06-04

**Authors:** Sajjaad H. Samat, Bradley Ruehle, Emily Sheldon, Hisham Qandeel

**Affiliations:** Department of Cardiothoracic Surgery, University of Michigan-Sparrow, 1215 East Michigan Avenue, Lansing, MI 48912 USA

**Keywords:** Mediastinal mass, Schwannoma, Spinal cord, Cardiothoracic, Cardiac, Tumor

## Abstract

Schwannoma is the most common neurogenic tumor in the posterior mediastinum, however it is rarely found in the anterior mediastinum (Mediastinum 4, 2020; Radiat Med 13(4):175-7). We report a large anterior mediastinal schwannoma causing cardiac compression along with a concurrent cervical spine lesion causing right-sided weakness. Current literature having only a few case reports describing an anterior location for this neoplasm and even rarer to have a concurrent symptomatic cervical lesion. We present this case to highlight this rare finding and increase awareness of this potential diagnosis.

## Case presentation

Our patient is a fifty-year-old male with a history of obesity and depression who was originally admitted after a syncopal episode, generalized weakness, and progressively worsening right arm and leg weakness.

**Fig. 1:**
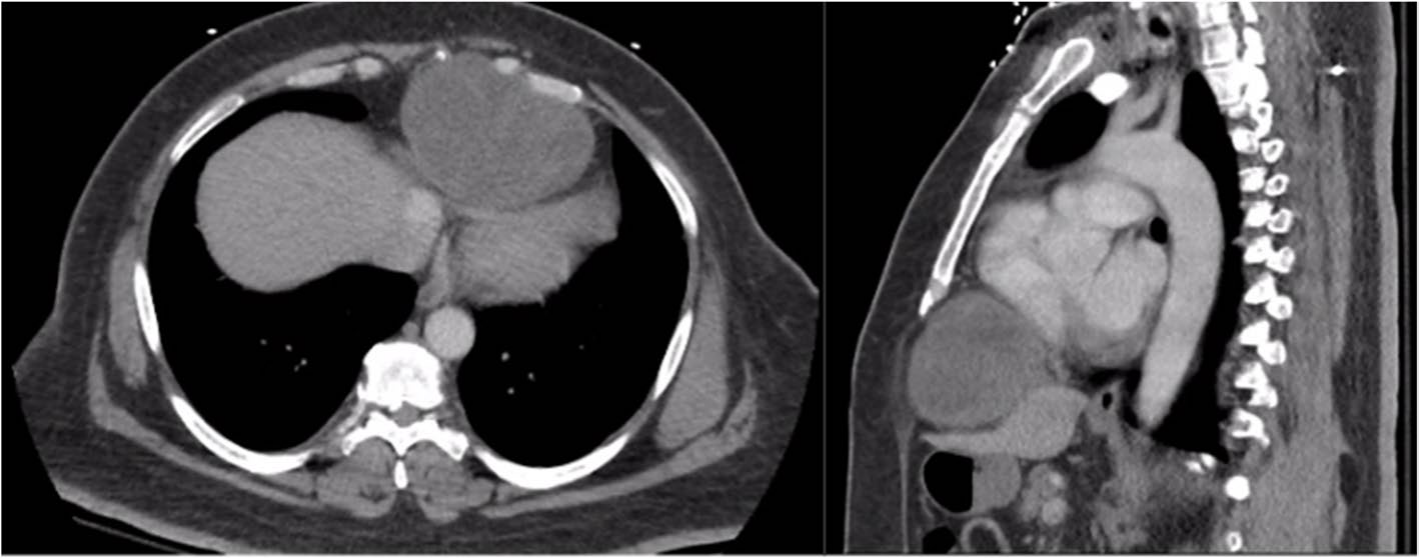
CT axial(left) and saggitial(right) chest and abdomen showing a large (10.6 x 7.2 x 8.3 cm) heterogeneous soft tissue mass in the mid/left paracentral region of the anterior lower thorax which abutted and caused mass effect upon the heart and diaphragm and partially extrudes at the level of the subxiphoid process

Workup included a computed tomography (CT) brain which was unremarkable. Further workup with a CT chest and abdomen showed a large (10.6 x 7.2 x 8.3 cm) heterogeneous soft tissue mass in the mid/left paracentral region of the anterior lower thorax (Fig. 1). The mass abutted and caused mass effect upon the heart and diaphragm. Transthoracic echocardiogram was obtained and showed this large soft tissue mass adjacent to and compressing the right ventricle.

**Fig. 2:**
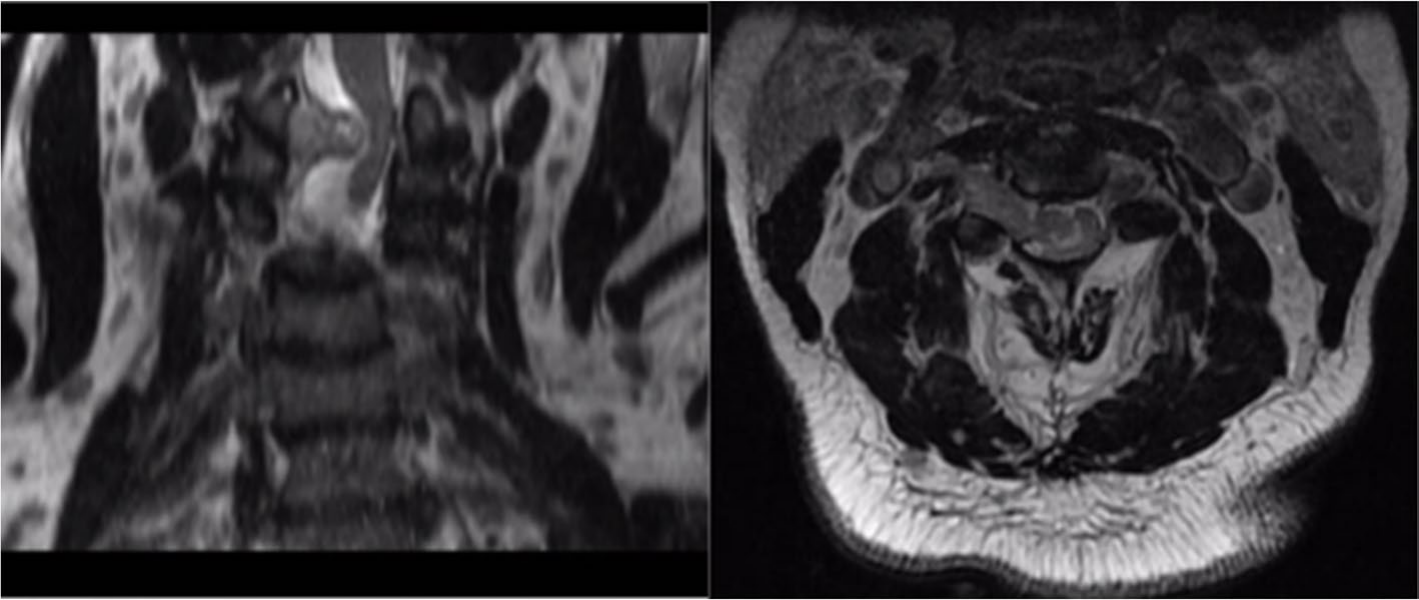
MRI showing (left and right T2 weighted cervical) spine was significant for an extradural 3-cm enhancing mass on the right at C2-C3 with severe compression of the cervical spinal cord and displacement of the cord into the left side of the canal, near to the vertebral artery

Given his neurological complaints and physical exam findings of right-sided weakness, magnetic resonance imaging (MRI) imaging was also obtained. MRI of the cervical spine was significant for an extradural 3 cm enhancing mass on the right at the C2-C3 level with severe compression of the cervical spinal cord and displacement of the cord to the left side of the canal (Fig. 2).

**Fig. 3:**
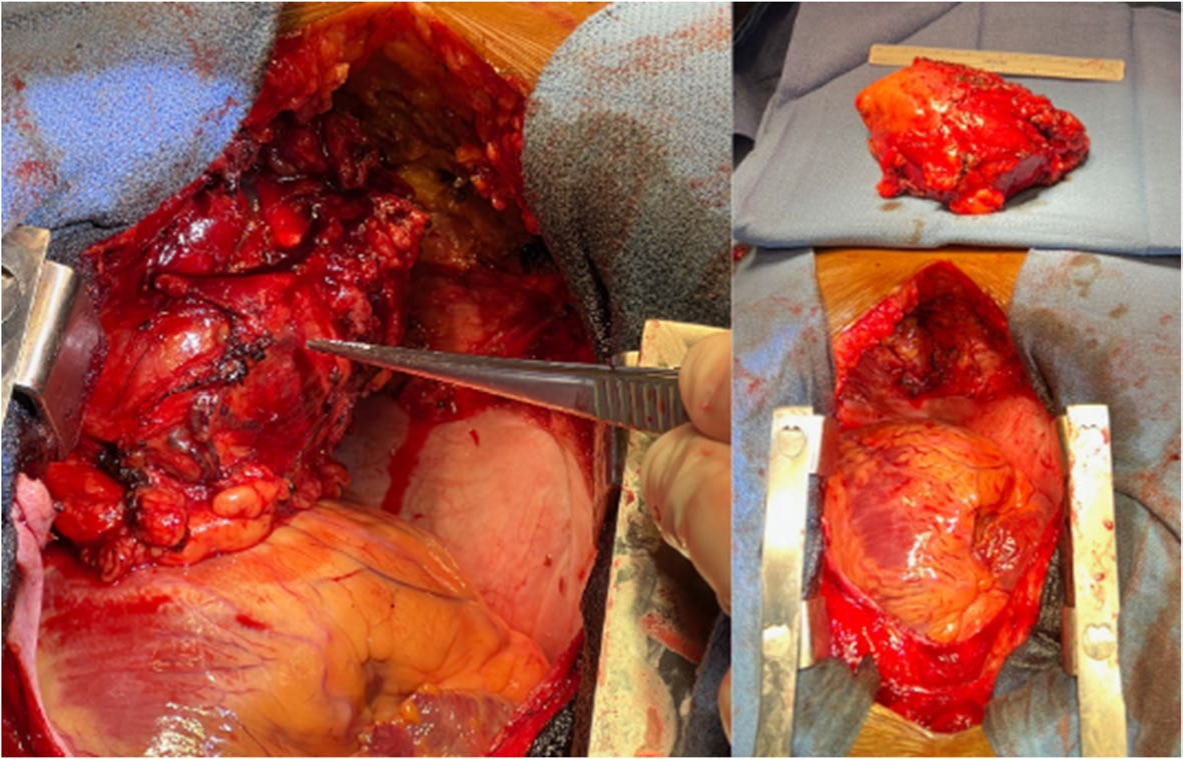
Left image showing anterior 12.5 x 8.5 x 6.0 cm mass in situ showing the degree of cardiac compression. Additional right image showing after mass excision and presentation on the backtable

Cardiothoracic surgery service was consulted and recommended surgical resection of the mediastinal mass for definitive diagnostic and therapeutic purposes. The patient and family wished to proceed with surgical resection. Patient underwent median sternotomy and had successful surgical resection of a 12.5 x 8.5 x 6.0 cm anterior mediastinal mass which was adherent to the pericardium, diaphragm, and found to be concurrently compressing the right ventricle (Fig. 3). Intraoperative frozen margins were negative and preliminary pathology showed suspected spindle cell neoplasm with concern for Schwannoma [1, 2]. Final pathology of the mass confirmed cellular Schwannoma with cystic and degenerative changes in addition to benign lymph nodes. Tumor cells were positive for S100 and OSX10, and negative for cytokeratin AE1/AE3, desmin, CD34, melanoma cocktail, p63, and STAT6.

Post-operatively the patient had an excellent recovery after his thoracic surgery and he was discharged to inpatient rehab on post operative day six. After a full recorvery from his thoracic resection surgery the patient subsequently underwent C2-C3 laminectomy and resection of cervical spine mass without significant postoperative complications. His right lower and upper extremity weakness and numbness drastically improved following this surgery and his main residual complaint was some pain in his right shoulder and right hand tightness.

## Discussion

The most common site of a mediastinal tumor is within the anterior mediastinum [3, 4]. Anterior mediastinal tumors commonly include: thymoma, teratoma, ectopic thyroid tissue and lymphoma. It is very rare to have neurogenic tumors located in the anterior mediastinum which are more commonly located in the posterior mediastinum [5].

The most common overall type of mediastinal tumor in both adults and children are neurogenic in nature and occur in the posterior mediastinum. Schwannoma is a type of mediastinal neurogenic tumor and is typically asymptomatic, however symptoms may occur due to the mass effect of the growing tumor (Marchevsky).

Schwannoma is a slow growing benign neural sheath tumor of the peripheral nervous system [6, 7]. Forty-five percent of schwannomas occur in the head and neck with only about nine percent occurring in the mediastinum(Kapoor). Mediastinal schwannomas most commonly occur from the sympathetic trunks or intercostal nerves in the paravertebral location; however, our patient was found to have an anterior schwannoma possibly originating from intercostal nerve branches in the anterolateral chest wall location.

**Fig. 4:**
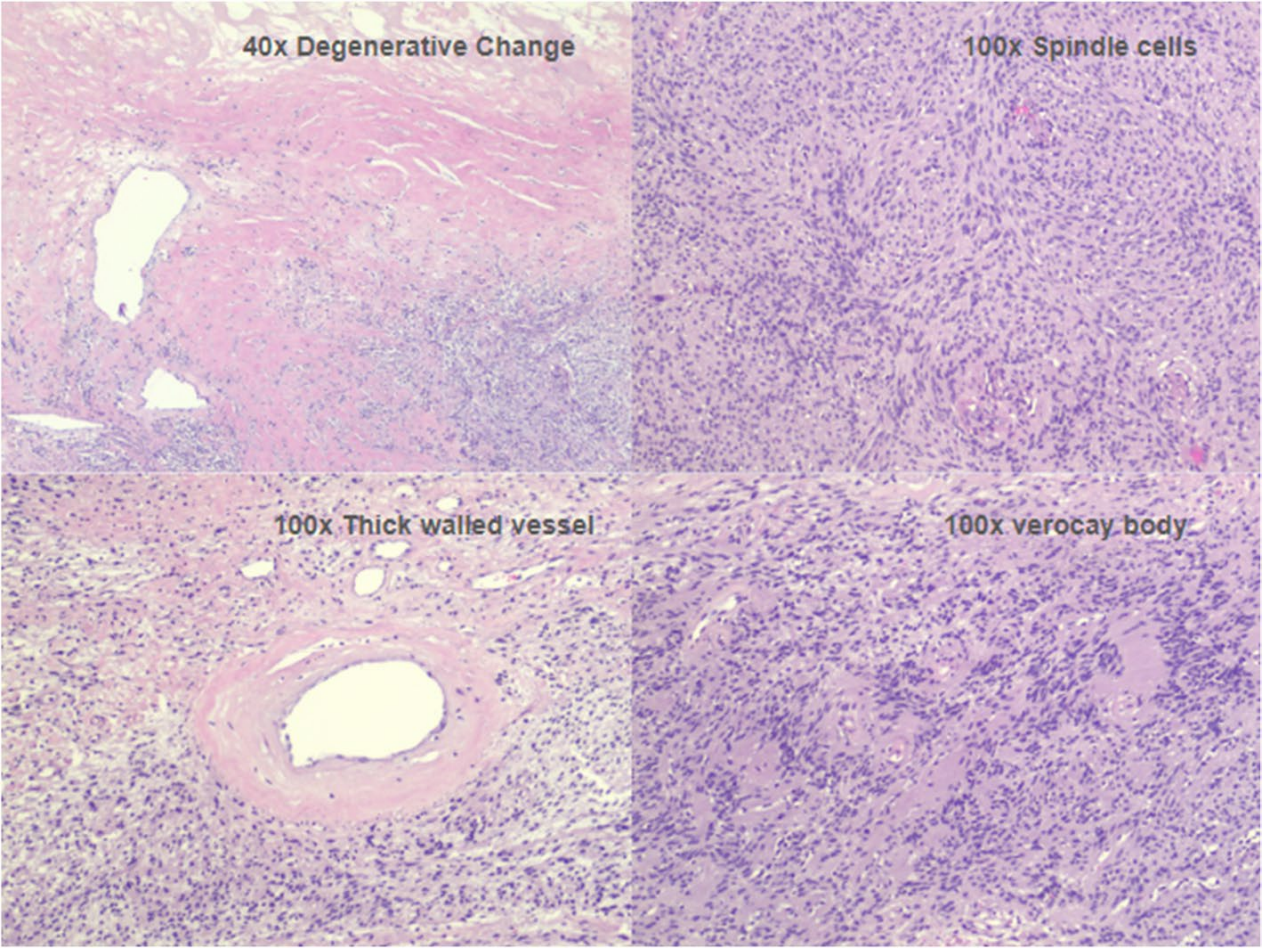
Histologic sections reveal a spindle cell neoplasm forming short fascicles, and admixed cellular Antoni A and hypocellular Antoni B. The tumor cells are bland with abundant eosinophilic cytoplasm. Verocay bodies are seen. Mitotic figures are not identified. Degenerative changes and thick-walled vessels are present

Histopathological features consistent with schwannoma showed positive S100 and OSX10, and negative for cytokeratin AE1/AE3, desmin, CD34, melanoma cocktail, p63, and STAT6 [8]. This finding is crucial for confirming the diagnosis and was consistent with the staining results obtained from our specimen (Fig. 4).

Our patient presents with a case of a symptomatic 12.5 x 8.5 x 6.0 cm anterior mediastinal mass with pathological stains consistent with Schwannoma. Symptomatology of the patient included syncope and right upper/lower extremity weakness. Excluding patients with neurofibromatosis, it is exceedingly rare to have concurrent lesions elsewhere. Our patient had a concurrent lesion in his cervical spine accounting for his motor deficits for which he would be seeking treatment in the future after recovery from his initial surgery. Anterior mediastinal schwannomas are rare, indolent in nature, and benign. However, they can lead to significant compressive symptoms of structures within the anterior mediastinum including airway or cardiovascular compromise as well as neurological deficits [9, 10]. This presentation poses an early diagnostic challenge and therefore our paper intends to share awareness for the development of high clinical suspicion of this disease process.

From our literature review no consensus on a standardized treatment modality has been proposed. The goal of this paper is to raise awareness of the rare anterior mediastinal tumor and to advocate for thorough investigation for concurrent lesions and recommend surgical resection of these masses to avoid potential complications.

## Data Availability

No datasets were generated or analysed during the current study.

## Abbreviations

CT: Computed Tomography
MRI: Magnetic Resonance Imaging

## References

[1] Dy P, Lajom C, Sanchez J. Middle mediastinal schwannoma concealed by asthma and GORD. *BMJ Case Rep*. 2018;2018:bcr2017223795. Published 2018 Mar 13. 10.1136/bcr-2017-223795 10.1136/bcr-2017-223795 PMC5878299 29535096

[2] Kim, Jin-Sung et al. Severe Spinal Cord Compression by Pure Giant Intradural Schwannoma of Cervical Spine. *Clin Neurol Neurosurg. 2018*. 110,17-19 10.1016/j.wneu.2017.10.106 29107727

[3] Wang J, Yan J, Ren S, Guo Y, Gao Y, Zhou L. Giant neurogenic tumors of mediastinum: report of two cases and literature review. Chin J Cancer Res. 2013;25(2):259–62. 10.3978/j.issn.1000-9604.2013.03.09. 23592909 10.3978/j.issn.1000-9604.2013.03.09 PMC3626982

[4] Albert AF, et al. Giant solitary cystic schwannoma of the cervical spine: a case report. Clin Neurol Neurosurg. 2012;114(4):396–8. 22104695 10.1016/j.clineuro.2011.10.039

[5] Almeida PT, Heller D. Anterior Mediastinal Mass. [Updated 2023 Feb 5]. In: StatPearls [Internet]. Treasure Island (FL): StatPearls Publishing; 2023. 31536215

[6] Marchevsky, Alberto M., & Bonnie Balzer. Mediastinal tumors of peripheral nerve origin (so-called neurogenic tumors). *Mediastinum* [Online], 4 (2020) 10.21037/med-20-43 PMC8794401 35118300

[7] Marchevsky AM. Mediastinal tumors of peripheral nervous system origin. Semin Diagn Pathol. 1999;16(1):65–78. 10355655

[8] Pekmezci M, Reuss DE, Hirbe AC, et al. Morphologic and immunohistochemical features of malignant peripheral nerve sheath tumors and cellular schwannomas. Mod Pathol. 2015;28(2):187–200. 10.1038/modpathol.2014.109. 25189642 10.1038/modpathol.2014.109 PMC6816504

[9] Kapoor A, Singhal MK, Narayan S, Beniwal S, Kumar HS. Mediastinal schwannoma: A clinical, pathologic, and imaging review. South Asian J Cancer. 2015 Apr-Jun;4(2):104-5. 10.4103/2278-330X.155708. PMID: 25992358; PMCID: PMC4418079. 10.4103/2278-330X.155708 PMC4418079 25992358

[10] Tajima H, Tajima N, Yamamoto K, Maeda S, Koizumi K, Kumazaki T, Yoshida H, Egami K. Anterior mediastinal schwannoma: a case report. Radiat Med. 1995;13(4):175–7. PMID: 8539444. 8539444

